# The impact of transient air pollution exposure on worker performance in Chinese soccer players

**DOI:** 10.1038/s41598-024-82322-w

**Published:** 2024-12-28

**Authors:** Ning Zhang, Xiahai Wei, Xuhui Huang, Pan He, Yunxiao Liu, Hongyou Liu, Tao Lin, Xin Shi, Zhu Liu, Richard B. Freeman

**Affiliations:** 1 Institute of Blue and Green Development, Shandong Univeristy, Weihai, China; 2 Department of Land Economy, University of Cambridge, Cambridge, UK; 3 School of Economics and Finance, Huaqiao University, Quanzhou, China; 4 Department of Earth System Science, Tsinghua University, Beijing, 100084 China; 5 SILC Business School, Shanghai University, Shanghai, 201899 China; 6 School of Physical Education and Sports Science, South China Normal University, Guangzhou, 510631 China; 7 College of Finance and Economics, Jimei University, Xiamen, China; 8 School of Health Management, China Medical University, Shenyang, 110122 China; 9 John F. Kennedy School of Government, Harvard University, Cambridge, 02138 USA; 10 Department of Economics College of Economics, Harvard University, Cambridge, 02138 USA

**Keywords:** Air pollution, Worker performance, Physical vs. cognitive ability, Soccer match, China, Environmental social sciences, Environmental economics, Environmental impact

## Abstract

Using a unique dataset on the performance of soccer players in China (retrieved from 632 matches involving 24 teams during the 2014 to 2016 seasons), we investigate the effect of air pollution on different performance indicators that rely on different mixtures of the physical and cognitive inputs of players. To ensure a causal interpretation, we implement an instrumental variable (IV) approach using thermal inversion as the instrument for air pollution. We found that players’ performance indicators, especially those more related to cognitive factors, are more strongly influenced by air pollution. One standard deviation (SD) increase in the Air Quality Index (AQI) leads to 2.5% decrease in the number of players’ passes and 5.1% increase in the number of fouls. However, for performance indicators that are more related to players’ physical condition, e.g., running distances, no such significant impact is identified. Overall, these findings suggest that the negative impact of short-term air pollution exposure on outdoor worker performance is mainly cognitive, which we believe could lead to important policy implications, not only for competitive sports but also across a much broader spectrum of business sectors.

**Supplementary Information:**

The online version contains supplementary material available at 10.1038/s41598-024-82322-w.

## Introduction

Researchers have shown that air pollutants severely injure lung and cardiovascular functions. They can also lead to significant cognitive damage due to their impact on blood flow and circulation^1–4^. Nowadays, air pollution has become a major environmental challenge to sustainable growth in the global economy. For example, it contributed to 5.5 million premature deaths in 2013, causing a loss of $225 billion in labor income worldwide^5^. Under these circumstances, a strand of literature investigates the consequences of short-term air pollution exposure on labor productivity and finds that it can reduce the performance of workers in both physically demanding and highly skilled positions^6–14^. While these studies have deepened our understanding of the indirect costs of air pollution, an important question remains: does the detrimental impact of short-term air pollutant exposure on worker performance rely more on its negative influence over physical condition or cognitive performance? This question is important for at least two reasons. First, it is directly related to the efficient allocation of valuable resources: should more resources be devoted to improving the cognitive ability of workers, or should their physical health conditions be of greater concern? Second, given the difference in productivity between physically and cognitively demanding workers in the modern economy, knowing which group is affected more strongly is important for a more precise evaluation of the indirect costs of air pollution. Note that a simple investigation of air pollution’s impact on physically (cognitively) demanding workers’ performance is insufficient to answer the question, since their performance can also be affected by cognitive (physical) factors. In this study, we provide the first piece of empirical evidence that compares the relative importance of these different mechanisms in a unique research setting.

Specifically, we focus on the performance indicators of professional soccer players. For four reasons, we believe this provides an ideal setting for the investigation of our research question. First and most importantly, soccer involves both cognitive skills and intensive physical activities. This feature enables us to identify multiple performance indicators that rely on differentiating mixtures of mental and physical inputs. For example, running distances are more likely to be determined by players’ physical health conditions^15–17^. Conversely, cognitive factors could play a relatively important role in performance indicators like the number of passes and fouls^18–24^. A detailed discussion of these indicators is available in the Online Appendix. Our study builds on and extends recent research, such as Li et al.^25^, which also investigates the relationship between air pollution and soccer performance. While both studies address short-term exposure and use an instrumental variable (IV) approach to mitigate endogeneity, Li et al.^25^ focus primarily on passing performance. In contrast, we adopt a broader approach by analyzing passes, fouls, and running distance. This multi-metric analysis allows us to distinguish more precisely between cognitive and physical impacts of air pollution.

Second, while previous studies primarily use within-individual variations (across different times) to identify the impact of transitory air pollution exposure^26^, one potential concern is that the generalizability of the results could be undermined if within-individual variations correlate with air pollution levels, since the within estimator is largely determined by individuals with larger within variations. Thus, it may only reflect a local effect for those constantly living in high air pollution locations (reflecting not only the short-term but also the long-term air pollution exposure). A unique feature in professional soccer enables us to identify a “clean” impact of short-term exposure and therefore alleviate the above concern: players must play away matches. This represents a sudden and transitory change in their exposure to air pollutants because players typically do not live in the cities where the matches take place.

**Fig. 1:**
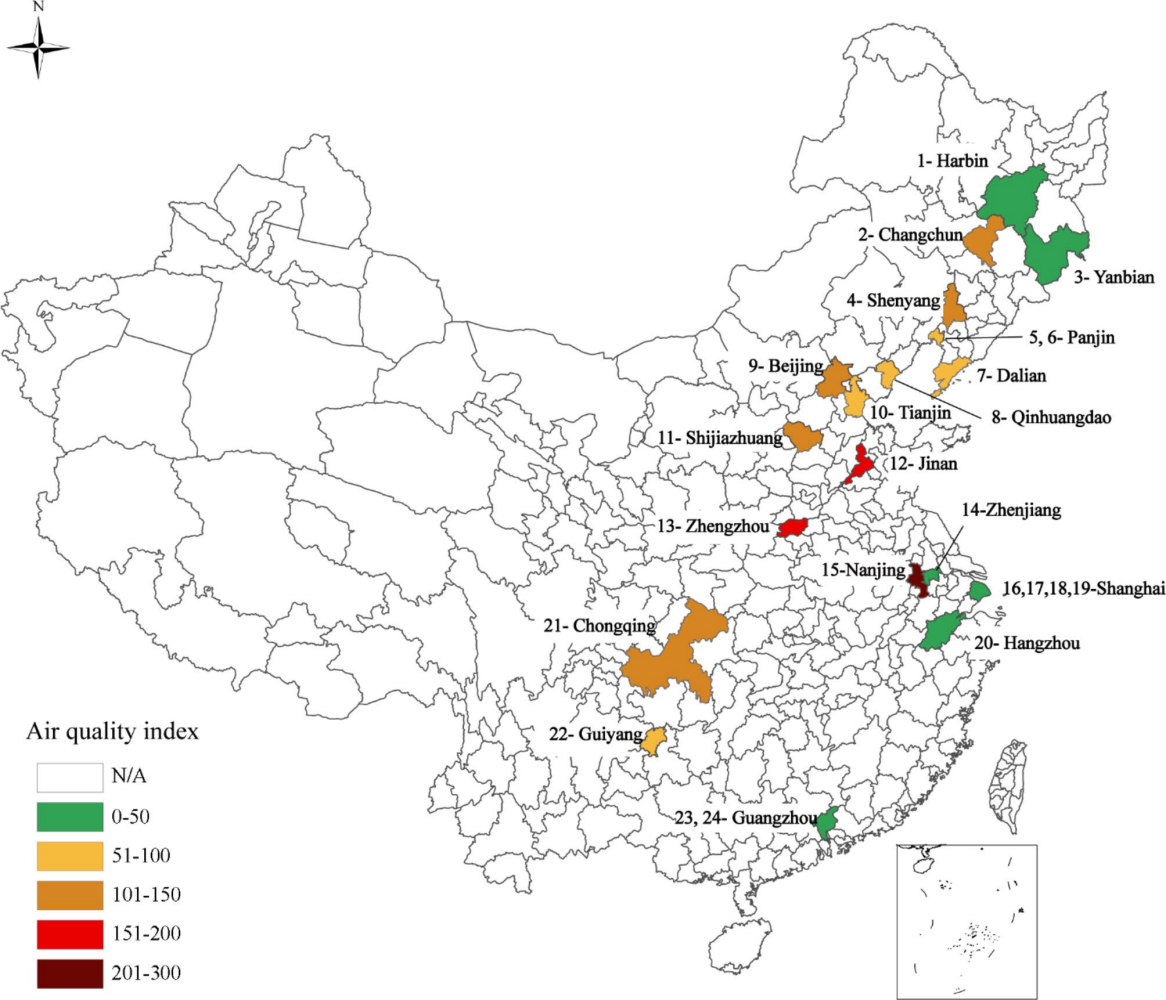
Match Location and Average AQI. This map shows the 16 cities (24 soccer stadiums) where the 632 matches in our sample took place, along with the average AQI level for each city during the sample period. The detailed list of the stadiums is given in Table A1.

Third, soccer players are directly exposed to air pollutants because all matches take place outdoors. The competitive nature of their work further increases the total airway exposure to pollutants, as their respiratory rate and volume increase substantially during the match. This helps us to identify the impact of air pollution, if any. Fourth, China is a country well-known for its air pollution problems^10–13^. Even in recent years, the average PM2.5 level has been shown to be about four times higher than that of most developed economies^27^, enabling us to examine the influence of severe air pollution. Additionally, the soccer matches we examined took place in 16 different cities scattered across the country. This ensures sufficient variation in air pollution levels for the identification of their effects (Fig. 1).

We focus on the Chinese Football Association Super League (CFASL), the highest tier of professional soccer in China, and collect players’ performance information from 632 matches (24 teams) during the 2014 to 2016 seasons. To address the endogeneity of air pollution and identify its causal effect on players’ performance, we implement an IV approach (The rationale for using the IV approach can be found in the Online Appendix). Specifically, we use thermal inversion, a complex meteorological phenomenon that is typically independent of economic activities, as the instument^11,28–30^. After controlling for player fixed effects (FE), identification is driven by the variation in the exogenous thermal inversion across different matches for the same player. Using a two-stage least squares (2SLS) estimator, we document a sizable impact of air pollution on players’ performance indicators, especially the ones that are more likely to be driven by cognitive factors: one standard deviation increase in the AQI leads to 2.5% decrease in the number of passes by the players. In addition to passes, poor air quality also leads the players to commit more fouls: one standard deviation increase in the AQI leads to 5.1% increase in the number of fouls. However, when we focus on players’ running distances, the indicator more reflective of their physical conditions, the impact of pollution disappears. This extraordinary difference suggests that the negative impact of short-term air pollution exposure on worker performance is mainly cognitive. We believe these findings are also applicable to other occupations that require both significant mental and physical inputs like soccer players, thus providing valuable policy implications for coping with severe air pollution.

## Results

### Impact of air pollution on Players’ cognitive skills

We first evaluate the validity of the instrument. The results in Table A4 show a strong first-stage relationship from the IV regressions, as evidenced by the statistically significant coefficient of thermal inversion. Additionally, the F-statistics are much larger than the 16.38 critical value^31^, suggesting thermal inversion is not a weak instrument.

Based on results from the second-stage IV regressions, we then evaluate the possible effect of air pollution on performance indicators that are largely driven by cognitive factors: i.e. number of passes and fouls. Panel A of Table 1 shows the results for passes. After controlling for time-varying player characteristics, flexible weather conditions, and player FE as well as home match FE, we observe a negative and statistically significant coefficient of AQI in Column (1). In Column (2), we additionally control for team and season FE to further capture the unobservable team factors and seasonal shocks. The coefficient of AQI remains significant, only slightly larger in magnitude compared to Column (1). Our baseline results are shown in Column (3) of Table 1, where we use a stringent setting by including Team*Season FE. These fixed effects account for the potential team-season specific shocks that could influence teams’ competing style since it is common for the composition of CFASL teams to change across different seasons (for example, due to coach turnover, or player transfer). Still, the coefficient of AQI is negative and statistically significant (-1.368). More importantly, if we calculate the economic significance of air pollution, we find that one SD increase in AQI leads to 2.5% decrease in the number of passes ((-1.368*0.509)/28.009), which we believe is a meaningful result. In the remaining columns of Table 1, we perform two robustness tests by replacing AQI with PM2.5 and PM10. The results are quite consistent: the coefficients of PM2.5 and PM10 are -1.916 and -0.957 respectively, both significant at the 5% level. As for economic significance, one SD increase in PM2.5 and PM10 leads to a reduction in the number of passes by 2.7% and 2.4% respectively, suggesting a larger impact of PM2.5. This observation is consistent with the findings in the scientific literature that PM2.5 causes greater damage to both physical and mental health because it can more easily penetrate deep into the body than PM10^32,33^.

**Table 1:** Impact of air pollution on soccer players’ performance: passes and fouls.

		Panel A: No. of Passes					Panel B: No. of Fouls			
	(1)	(2)	(3)	(4)	(5)	(6)	(7)	(8)	(9)	(10)
AQI	-1.195**	-1.279**	-1.368**			0.106*	0.108*	0.121**		
	(0.564)	(0.548)	(0.548)			(0.059)	(0.058)	(0.059)		
PM2.5				-1.916**					0.153*	
				(0.777)					(0.084)	
PM10					-0.957**					0.093**
					(0.409)					(0.043)
Player and Weather Controls	Y	Y	Y	Y	Y	Y	Y	Y	Y	Y
Player FE	Y	Y	Y	Y	Y	Y	Y	Y	Y	Y
Home FE	Y	Y	Y	Y	Y	Y	Y	Y	Y	Y
Team FE	N	Y	N	N	N	N	Y	N	N	N
Season FE	N	Y	N	N	N	N	Y	N	N	N
Team*Season FE	N	N	Y	Y	Y	N	N	Y	Y	Y
Observations	15,061	15,061	15,061	15,009	14,227	15,061	15,061	15,061	15,009	14,227

“FE” stands for fixed effect

This table reports the results of air pollution’s impact on player’s passes and fouls from the second stage IV estimation. Columns (1)-(5) show the results for the number of passes while Columns (6)-(10) show the results for the number of fouls. The independent variables are AQI (Columns 1–3, 6–8), PM2.5 (Columns 4, 9), and PM10 (Columns 5, 10). We control for player characteristics (Age, Playtime, Defender Indicator, Midfield Indicator, and Forward Indicator) and weather variables (Temperature, Dewpoint, Humidity, Wind Speed, and Cloudy Day Indicator). Standard errors are clustered at the player-level and reported in parentheses. * p < 0.1, ** p < 0.05, *** p < 0.01.

Panel B of Table 1 shows the results for fouls. We find that the coefficients of AQI all enter positively across different specifications (Columns 1–3). In terms of economic significance, our baseline regression results (Column 3) suggest that one SD increase in AQI leads to 5.1% increase in fouls, an even larger impact than with the number of passes. Similarly, when we focus on PM2.5 and PM10, the results are quite consistent: both coefficients are positive and statistically significant, while the magnitude is larger for PM2.5.

### Heterogeneity across different positions

Overall, the results in Table 1 suggest that air pollution has an economically meaningful impact on soccer players’ cognition-related skills such as passes and fouls. We explore the heterogeneous effect of air pollution across different positions: defenders, midfield, and forward, to better understand the underlying factor through which air pollution affects player’s performance. The sports science literature shows that defense players on average have the highest number of passes and the lowest numbers of fouls, the two performance indicators that represent superior cognitive ability, followed by midfielder and forward^34^. Thus, we expect the negative impact of air pollution to be the strongest for defenders.

**Fig. 2:**
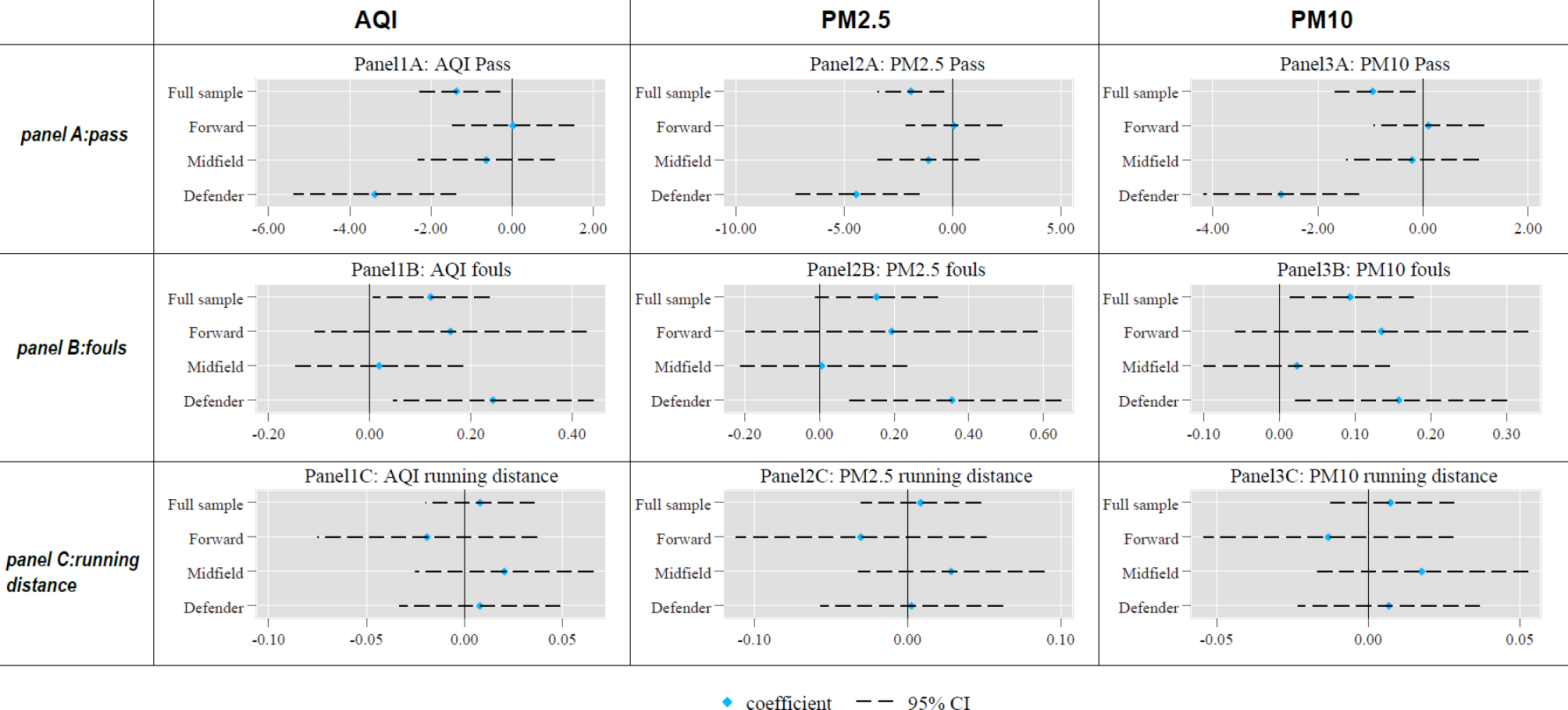
The Heterogenous Impact of Air Pollution across Different Positions. The figures plot the estimated coefficients on air pollution (AQI, PM2.5, and PM10) for different position subsamples (Defender, Midfielder, and Forward) with 95% confidence intervals based on the estimates in Tables A7 and A12. The full sample results are also reported as the baseline. Panel A, B, and C refer to number of passes, number of fouls, and running distances, respectively.

The second-stage IV regression results in Table A7 confirm our conjecture (the results are visually presented in Fig. 2 as well). In Panel A, the coefficient of AQI for defenders is -3.379, significant at the 1% level. This means one SD increase in AQI will lead to 5.9% reduction in defenders’ number of passes, twice larger than the full sample estimate. Additionally, if we focus on the magnitude-based inference (MBI) approach^35^, a statistical analysis method commonly used in sports science, the results remain meaningful. The results from the standardized regressions (Table A8) show that two SD change in AQI leads to 0.222 SD change in the defenders’ number of passes. This constitutes a small yet non-trivial impact according to the MBI framework (< 0.2 trivial; 0.2–0.6 small; 0.6–1.2 moderate; 1.2–2.0 large; > 2.0 very large), which is reasonable given that air quality only represents a small portion of the determining factors for a player’s performance. In terms of uncertainty, the results (Figure A1) show that AQI is likely to have a negative impact on defenders’ number of passes. If we replace AQI with PM2.5 and PM10, the results are quite consistent, as shown in Columns (4) and (7): their coefficients are negative and highly significant, with the economic magnitude of PM2.5 larger than that of PM10.

If we move from defenders to midfielders and then forwards, the impact of air pollution diminishes monotonically. As shown in Columns (2), (5), and (8), the coefficients of AQI, PM2.5, and PM10 are smaller in magnitude for midfielders and not statistically significant. For forwards, even the signs of the coefficients are flipped with the absolute magnitude indistinguishable from zero.

Panel B of Table A7 reports results for the number of fouls. Consistent with the results in Panel A, the effect of air pollution on fouls is observed primarily for defenders: one SD increase in AQI leads to 9.6% increase in defenders’ number of fouls. For midfielders and forwards, the coefficients of the three different pollution measures are not statistically significant, although their magnitudes are larger for the forward subgroup.

### Heterogeneity between home and away matches

**Fig. 3:**
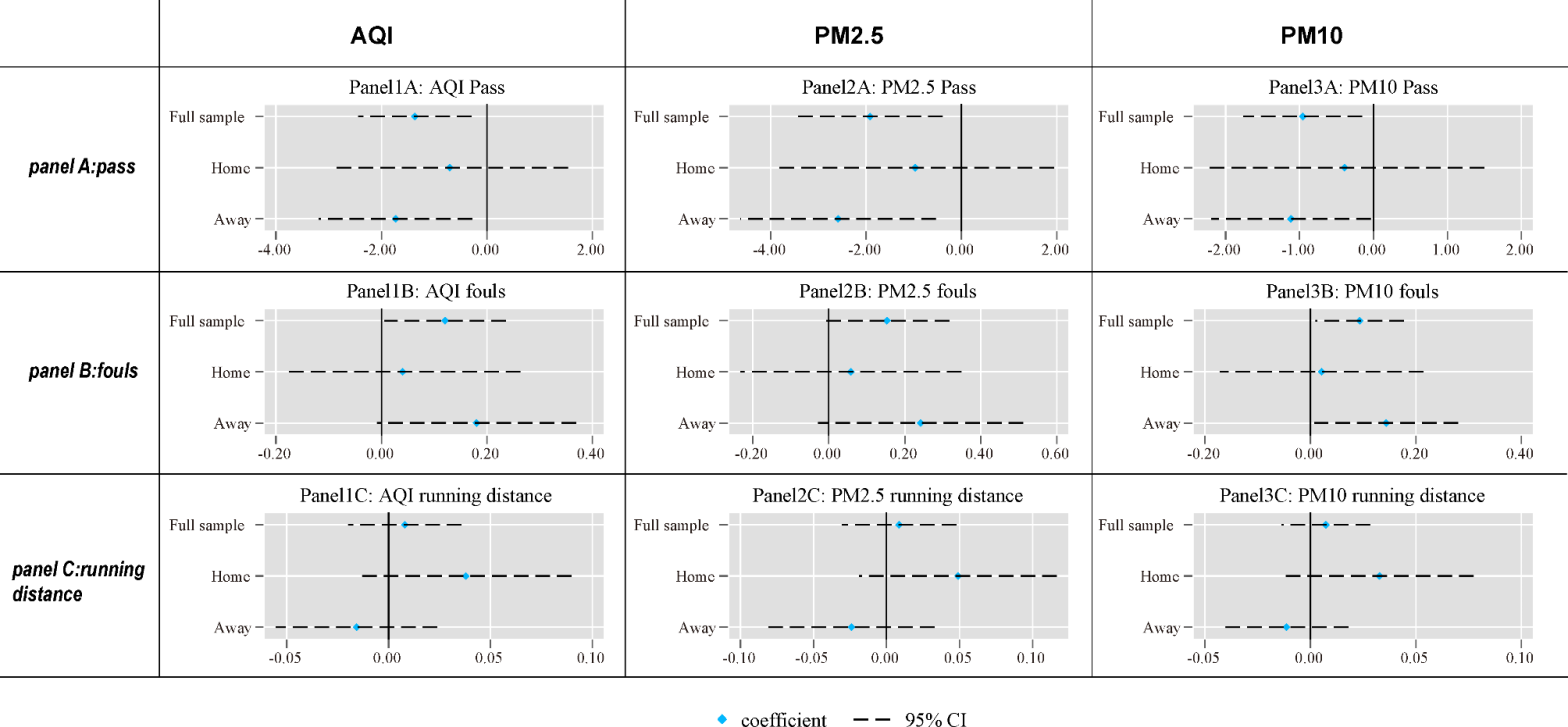
The heterogenous impact of air pollution b/w home and away matches. The figures plot the estimated coefficients on air pollution (AQI, PM2.5, and PM10) for home and away matches with 95% confidence intervals based on the estimates in Tables A9 and A12. The full sample results are also reported as the baseline. Panel A, B, and C refer to number of passes, number of fouls, and running distances, respectively.

To further tease out the short-term impact of air pollution, we divide our sample into home and away matches. If the detrimental impact of air pollution we identified earlier is due to short-term exposure, we should observe significant results for away matches. Since these players do not live in the cities where the matches take place, they are only exposed to severe air pollution for a short time. The results in Fig. 3 and Table A9 confirm our prediction. For away matches, the coefficients of AQI, PM2.5, and PM10 in Panel A are all significant (at the 5% level) and are larger in magnitude than the full sample results in Table 1. This suggests that short-term exposure to air pollution indeed reduces the number of passes. In terms of economic significance, one SD increase in AQI, PM2.5, and PM10 will lead to 3.1%, 3.7%, and 2.8% decrease in the number of passes, respectively. If we move to Panel B, we observe a similar pattern for the number of fouls: away team players experience a statistically significant increase in the number of fouls when facing higher air pollution levels, and the coefficients of different air pollution indicators are all larger than those of their full sample counterparts.

However, if we look at the results for home team players, the coefficients are small in magnitude and are not significant. One explanation for this observation is that the impact of air pollution on cognitive performance is only temporary, since players on the home team exposed to high-level air pollution for a long period of time may become adapted to it. An alternative explanation is that the advantage of playing at home may offset the adverse impact of air quality on players’ performances (commonly known as home advantage).

### Non-linearity in air pollution’s impact

The literature suggests that the substantial negative effects of air pollution might not emerge until pollution levels exceed certain thresholds^12,36,37^. To verify whether this is true for players’ performances, we first use Kernel-weighted local polynomial smoothing, a nonparametric estimation, to investigate the relationship between different levels of air pollution measures and the two performance indicators, without any further controls^8^. Based on the results, the different threshold levels of air pollution (AQI, PM2.5, and PM10) that we will use in the formal analysis are determined. For example, the two thresholds for AQI are 40 and 160 when the outcome variable is the number of passes. They change slightly to 50 and 160 when the number of fouls is the variable of interest.

We then formally test whether the nonlinear effects of air pollution apply to our current model framework. Specifically, we generate two sets of air pollution dummies based on the different threshold levels uncovered earlier and replace the original air pollution measures with these dummies in the regressions (Here we do not implement the IV approach since the number of endogenous variables exceeds that of the instrument.). Table A11 shows the varying impact of air pollution: in Column (1), the coefficient of Intermediate Pollution Level (40 < AQI <  = 160) is -0.562 and statistically significant at the 1% level, suggesting that the incremental effect of AQI is significant as it moves from low to intermediate level. When the AQI exceeds the threshold of 160, its impact becomes larger, as evidenced by the significant coefficient of -0.742. The results for the number of fouls are similar, albeit weaker in statistical significance (Column 4). In sum, we observe a monotonically increasing pattern of the impact of air pollution as it moves from a lower to a higher level. For the other two air pollution measures, PM2.5 (Columns 2 and 5) and PM10 (Columns 3 and 6), the results are quite consistent.

### Impact of air pollution on players’ physical performance

After confirming the negative influence of air pollution on players’ cognitive performance, we change our focus to performance indicators that are more influenced by players’ physical condition, i.e. running distances. We perform the same set of IV regression analyses using running distance as the dependent variable. The results are reported in Table 2. In all specifications, the coefficients of air pollution levels (measured by AQI, PM2.5, or PM10) never reach significance. More importantly, their magnitudes are close to zero (for example, only 0.008 for the baseline regression in Column 3). In Table A12, we also performed additional analyses focusing on different players’ positions, home versus away matches, and the potential non-linear effect. The coefficients of pollution levels are almost always insignificant and small in magnitude. We also fail to identify a clearly monotonic pattern of air pollution influence as it moves from low to high level. These results, taken together, suggest that the impact of short-term air pollution exposure on physical performance is negligible. Thus, the negative impact of air pollution on outdoor worker performance mainly comes from the cognitive channel.

**Table 2:** Impact of air pollution on soccer players’ performance: running distances.

	Running distances				
	(1)	(2)	(3)	(4)	(5)
AQI	0.012	0.013	0.008		
	(0.014)	(0.014)	(0.014)		
PM2.5				0.009	
				(0.020)	
PM10					0.007
					(0.011)
Player and Weather Controls	Y	Y	Y	Y	Y
Player FE	Y	Y	Y	Y	Y
Home FE	Y	Y	Y	Y	Y
Team FE	N	Y	N	N	N
Season FE	N	Y	N	N	N
Team*Season FE	N	N	Y	Y	Y
Observations	15,058	15,058	15,058	15,006	14,224

“FE” stands for fixed effect

This table reports the results of air pollution’s impact on player’s running distances from the second stage IV estimation. The independent variables are AQI (Columns 1–3), PM2.5 (Column 4), and PM10 (Column 5). We control for player characteristics (Age, Playtime, Defender Indicator, Midfield Indicator, and Forward Indicator) and weather variables (Temperature, Dewpoint, Humidity, Wind Speed, and Cloudy Day Indicator). Standard errors are clustered at the player-level and reported in parentheses. * p < 0.1, **p < 0.05, ***p < 0.01.

### Impact of air pollution on match-level outcomes

We investigate whether the negative impact of air pollution we discovered earlier accumulates to affect match outcomes. We thus focus on match-level data and compare the differences between away and home teams, since their players’ performances are affected differently. Table A13 shows the results for game scores from the second-stage IV regressions. If we look at goals scored, the coefficients of the interaction terms between different air pollution measures and the away team indicator are all negative and statistically significant. Taking AQI as an example, the coefficient of -0.843 suggests that 100-point increase in AQI (roughly two SD change) will reduce away team goals by 0.843 goals scored, compared to home teams. When we focus on the number of goals conceded, the coefficients of the interaction terms become positive and significant, suggesting that away teams are more likely to be scored on than home teams. Not surprisingly, goal difference, the indicator that is directly related to match outcomes, is also significantly different between away and home teams as air pollution intensifies.

Next, we directly examine the impact of air pollution on winning probabilities. Columns (1)–(3) in Table A14 show the second-stage results from the linear probability models in which losing the game equals zero, and one otherwise. For example, the coefficient of the interaction term between AQI and the away indicator is -0.407 in Column (1). This result indicates that 100-point increase in AQI will reduce the winning probability of the away teams by 40.7% compared to home teams, which we believe is a fairly large amount. In the last three columns, the results remain consistent using probit models. To further alleviate the concern that we treat win and tie equivalently in the binary outcome model, in Table A15 we implement an ordered probit model in which lose, tie, and win equal zero, one, and two, respectively. Again, the interaction terms between air pollution and the away indicator are negative, suggesting that our results are not influenced by specific model choices.

Overall, the results show that the deterioration in player performance caused by short-term air pollution exposure has real consequences, in the sense that it substantially lowers the probability of winning by away teams.

## Discussion

Our findings have several important implications for decision-makers. First, our results suggest that players’ cognition-related performance (like passes and fouls) is significantly affected by air pollution: one standard deviation increase in the AQI leads to 2.5% decrease in the number of passes and 5.1% increase in the number of fouls. This impact is non-trivial for certain positions (defenders) even based on the MBI framework, the methodology commonly used in sports science. One direct implication for the management and coaching teams is that they should devote more resources to the training of their players’ cognitive skills. This is especially important for teams facing away matches: tactical adjustment should be made and certain forms of special training that target cognitive abilities should be performed before competing in a stadium exposed to high levels of air pollutants, because the winning probability of away teams is severely reduced by degraded air quality. By contrast, we do not find any evidence of deterioration in physical performance indicators (like running distances) caused by air pollution. This finding, albeit a surprising one, may suggest that physical training, if necessary, is still feasible during hazy days.

In addition to individual teams, our findings are also important for the football association, the governing body of professional soccer in China. The results show that playing under severe air pollution conditions significantly reduces the relative performance of away teams: compared to home teams, 100-point increase in the AQI reduces the net scores of the away teams by 1.537 and the winning probability by 40.7% on average. These findings indicate that air pollution exposure could be a non-negligible obstacle for the overall fairness of the matches. One possible solution to this problem is to move matches to indoor stadiums in highly polluted cities. While our findings do not mean this solution, which is obviously not costless, is necessary, a careful evaluation of its costs and benefits would clearly be beneficial.

Finally, we found that cognitive-related work performance is more severely affected by short-term air pollution exposure. This finding has implications for a much broader swath of the economy. It is well accepted that occupations that require intensive cognitive skills (attention, memory, logic and reasoning, auditory and visual processing) are critical for the growth of the modern economy, especially for developing economies like China. Given that our study focuses on professional athletes, the observed minimal impact of air pollution on physical performance may, in part, result from the “healthy worker effect”. Athletes, due to their rigorous physical training and heightened respiratory and cardiovascular fitness, may exhibit stronger physiological resilience against pollution exposure compared to the general population. This suggests that our findings on physical performance may not fully generalize to ordinary workers, particularly those with preexisting health conditions or lower baseline physical fitness. Our findings thus suggest that the cost of air pollution may be even higher than previously thought, and the potential benefits from improving air quality are even larger. However, given that the new national air quality standard is so costly, some cost-effective measures might be necessary in the short run to prevent workers, especially those relying on massive cognitive skills (such as researchers, analysts, professors, and government officials, among others), from being exposed to air pollutants. These might include wearing PM2.5 protective masks in outdoor working environments and installing air purification and filtration systems in indoor workplaces. Additionally, more time and effort could be devoted to improving workers’ mental health conditions in facing high air pollution.

While we believe this study deepened our understanding of the detrimental impact of air pollution on worker performance, it is not without limitations. We conclude by pointing out two such limitations and thus directions for future research. First, our study focuses solely on the cost of air pollution (or the benefit side of counter-air pollution policies). A rigorous cost–benefit analysis with a specific focus remains necessary for the implementation of such policies. Second, our study focuses on the short-term impact of air pollution on workers’ cognitive performance, while the potential effect of long-term air pollutant exposure remains unknown. For example, whether the detrimental impact cumulates as people are exposed to high-level air pollution for a long time or the impact is only temporary as they become more adapted and make adjustments mentally. The answers to these questions are of critical importance for us to understand the effect of air pollution more completely.

### Data and methods

#### Data sources

The information of CFASL matches and players comes from the NetEase super data live broadcast system provided by a unique database. The CFASL matches are organized by the Chinese Soccer Association (CFA) and are the highest level of professional soccer in China. The schedule is determined by the CFA and CSL League Limited liability company, including the date and kick-off time for each match. Each team selects a regular stadium in the city as its home ground and another stadium in the same city as a backup. Then, the matches are played in the manner of a double circle of home and away games, with 30 rounds and 240 matches throughout the season in total. We collect the data in the playing seasons during the period of 2014–2016. Information on matches includes their field, date, kick-off time, and whether it is a home or away match, while information on players includes their age, nationality, position (goalkeeper, defender, midfielder, or forward), playing time, steals, shovels, dribbling, passes, fouls, assists, shots, and running distances.

Data on air quality come from the real-time ground station monitoring of air quality provided by the National Environmental Monitoring Center of China (http://webinterface.cnemc.cn/). The monitoring data includes the hourly concentration of key pollutants including PM10, PM2.5, SO_2_, NOx, O_3_, and CO. Based on such concentration records, the AQI is also calculated as a nonlinear dimensionless index that quantitatively and comprehensively measures air quality (a detailed methodology for AQI calculation is introduced in a previous study^32^). In our sample, each match venue (shown in Table A1) is matched with the nearest monitoring station, with the venues linked to daily average air quality level.

The meteorological records come from weather underground (www.wunderground.com). This website provides real-time weather information sourced from services provided by national and personal ground stations. Hourly records for multiple variables are available, including temperature, precipitation, dew point, wind speed, visibility, pressure, and weather conditions (cloudy, rain, snow, clear, etc*.*). Similar to air quality data, we conduct a nearest neighbor match and use the daily average data for the empirical analysis.

The thermal inversion data is taken from MERRA-2. This dataset reports air temperature for each 50*60-km grid for different atmospheric layers. The data are available at 6-h periods from 1980 onwards. We aggregate all data from grid to city. We determine the existence of a thermal inversion if the temperature in the second layer is higher than that of the first layer for each 6-h period^38^.

### Empirical strategies

This study aims to estimate the impacts of air quality on short-term outdoor player’s performances via multiple pathways using data on the numbers of passes, fouls, and running distance by players in CFASL matches. Since performance in soccer games relies on perceptual-cognitive skills as well as physical strength, we involve multiple dependent variables to test the effect of air pollution on the dependent variables. The number of passes and the number of fouls is selected to measure the perceptual-cognitive skills, while running distance is chosen to indicate performance that leans more on physical strength. The air quality is measured by the local AQI. Since PM2.5 and PM10 are the major pollutants in China, we also include regressions on them as robustness check.

We estimate the following 2SLS model to examine the causal effect of air pollution on the performances of the players using Stata Version 16:

1 $$\begin{aligned}Performance_{{imt}} &= \beta _{0} + \beta _{1} Air~Pollution_{{mt}} + {\mathbf{Z}}_{\varvec {imt}} \\ &\quad + {\mathbf{W}}_{\varvec{mt}} + \gamma _{i} + h_{i} + Team_{i} \times Season_{m} + \upvarepsilon _{{im}}\end{aligned}$$

2 $$\begin{aligned}{Air~ Pollution}_{mt}&={\alpha }_{0}+{\alpha }_{1}{Thermal Inversion}_{mt}+{\mathbf{Z}}_{{\varvec{imt}}}\\ &\quad +{\mathbf{W}}_{{\varvec{m}}{\varvec{t}}}+{\gamma }_{i}+{h}_{i}+{Team}_{i}\times {Season}_{m}+{\upmu }_{im}\end{aligned}$$

where the subscript i represents an individual player, m indicates the playing venue, and t denotes the match date, respectively. In (1), the dependent variable,${Performance}_{imt}$, refers to the performance indicators of players, including number of passes, fouls, and the running distance in venue m at date t. ${Air Pollution}_{mt}$ denotes the air quality level of the station that is nearest to venue m at the match day t, measured by AQI, PM2.5, and PM10, respectively. ${\mathbf{Z}}_{{\varvec{i}}{\varvec{m}}}$ indicates a series of time-varying characteristics of individual players, including age, the quadratic term of age, playing time, and position (defender, midfielder, or forward).${\gamma }_{i}$ refers to a player FE that controls for time-invariant individual characteristics, such as skill and playing style, ensuring that personal differences do not bias our results. ${h}_{i}$ denotes the home match fixed effect, controlling for advantages inherent to playing at home. ${\mathbf{W}}_{{\varvec{m}}{\varvec{t}}}$ controls meteorological variables include temperature, the quadratic term of temperature, humidity, dewpoint, cloudy dummy, and wind speed at match day t. We also include the Team*Season FE, to address performance variations across seasons driven by changes in team composition, management, or tactics. ${\varepsilon }_{im}$ and ${\upmu }_{im}$ is the random error term. In (2), we instrument ${Air ~Pollution}_{mt}$ using ${Thermal ~Inversion}_{mt}$ denoting whether a temperature inversion existed in the match day t. We also control for other factors of temperature inversion, including the number of thermal inversions and their strengths in the specifications.

## Electronic supplementary material

Below is the link to the electronic supplementary material.

Supplementary Material 1

## Data Availability

The datasets used and/or analysed during the current study available from the corresponding author on reasonable request.
