# Supplementary material for: The impact of transient air pollution exposure on worker performance in Chinese soccer players
Source: Sci Rep. 2024 Dec 28;14:31093. doi: 10.1038/s41598-024-82322-w (PMC11680904; doi:10.1038/s41598-024-82322-w)
Supplement: Supplementary file 1 — Supplementary Material 1 [file 41598_2024_82322_MOESM1_ESM.docx]

**The impact of transient air pollution exposure on worker performance in Chinese soccer players**

**Online Appendix**

**Discussion on the selection of performance indicators**

In this study, we select running distance, passes, and fouls as key performance indicators to distinguish between the physical and cognitive effects of air pollution on soccer players. The rationale for using these specific indicators is grounded in the distinct nature of cognitive and physical demands required in soccer performance.

In sports science, running distance is widely accepted as a reliable measure of physical fitness and stamina in soccer (Carling, 2013; Carling & Dupont, 2011; Dolci et al., 2020; Gregson et al., 2010). It reflects a player’s ability to sustain high-intensity physical activities throughout a match, directly tied to their aerobic capacity and physical conditioning. Players with superior endurance are more likely to cover greater distances during competitive play. For instance, Carling & Dupont (2011) highlight that key components of physical performance include the total distance covered, the distance covered at high speeds, and the distance covered with ball possession. These metrics capture not only physical exertion but also tactical involvement, reflecting a player’s stamina and fitness under demanding conditions.

In contrast to running distance, passes and fouls are more strongly associated with cognitive skills such as decision-making, situational awareness, and emotional regulation (Ali, 2011; Gabbett et al., 2008; Romeas et al., 2016). Passing involves processing complex information in real-time – anticipating the positions of teammates and opponents and executing accurate passes under pressure. Ali (2011) highlighted that passing proficiency reflects a player’s capacity to perceive and react quickly to complex game scenarios. Romeas et al. (2016) further demonstrated that training cognitive functions improve passing accuracy, reinforcing the cognitive nature of passing as a performance metric. Thus, passing serves as an effective indicator of cognitive performance, particularly in dynamic, high-pressure match environments.

Fouls also provide insight into players’ emotional regulation and cognitive control. Research shows that cognitive overload and emotional stress can impair judgment, increasing the likelihood of fouls (Junge et al., 2000; van Maarseveen et al., 2016; Volkamer, 1971). For example, Volkamer (1971) observed that players from trailing teams are more prone to committing fouls due to frustration. Similarly, away teams often commit more fouls, likely influenced by crowd pressure from home spectators. Junge et al. (2000) found that players with poor anger management are more prone to fouling, suggesting that fouls are indicative of cognitive strain – especially in situations where emotional control is critical. Together, passes and fouls reflect the cognitive demands of soccer performance, making them suitable indicators for assessing cognitive impairment under air pollution exposure.

**Rationale for the IV approach and choice of thermal inversion**

In this study, we adopt an instrumental variable (IV) approach to address potential endogeneity concerns and accurately estimate the causal effect of air pollution on player performance. The use of an IV approach is crucial because of several challenges inherent in measuring the impact of environmental stressors.

*1. Addressing endogeneity in air pollution studies*

Air pollution is influenced by various economic activities and environmental factors, making it susceptible to endogeneity issues. Specifically, two sources of endogeneity are particularly relevant: (1) Reverse causality. Large-scale sports events attract significant crowd movement and traffic, which can increase local pollution levels (Locke, 2019; Wicker, 2018, 2019). Wicker (2018, 2019) explored the carbon footprint of stakeholders such as spectators and participants, showing how sport events can increase pollution. Locke (2019) further found that attendance at Major League Baseball games had a measurable impact on local air quality. This raises reverse causality concerns – pollution levels can be affected by the very events whose outcomes we aim to study, complicating causal inference. (2) Omitted variable bias and measurement error. Air pollution data are typically collected from monitoring stations that may not be situated close to sports venues, which introduces measurement error (Deryugina et al., 2019). Moreover, unobserved local factors, such as concurrent events or traffic variations, may confound the relationship between pollution and performance if not properly accounted for. These challenges make a robust identification strategy essential.

Given these challenges, a standard ordinary least squares (OLS) approach would yield biased estimates. Therefore, we implement an IV approach to address these concerns by introducing an exogenous source of variation in air pollution levels.

*2. Thermal inversion as an instrumental variable*

We use thermal inversion, a meteorological phenomenon independent of human behavior, as an IV to introduce exogenous variation in pollution levels. Thermal inversion occurs when a layer of warm air traps pollutants near the ground, reducing vertical circulation and increasing pollution concentrations. This phenomenon provides an ideal instrument for several reasons: (1) Exogeneity from economic activities. Thermal inversion is determined by atmospheric conditions rather than human activities (Arceo et al., 2016). This ensures that pollution levels resulting from thermal inversion are unrelated to soccer matches or team performance, making it a valid instrument. (2) Robustness and validation in existing studies. Thermal inversion has been used as an IV in multiple studies to mitigate endogeneity arising from reverse causality and measurement errors (Fu et al., 2021; Godzinski & Castillo, 2021; Wan et al., 2024). While soccer events are not as pollution-intensive as industrial activities, thermal inversion remains a valid instrument for identifying exogenous variations in pollution that affect athletes’ performance. (3) Controlling for ground-level weather patterns. While thermal inversion increases pollution concentration, it may coincide with local weather changes, such as temperature shifts. To ensure that observed performance changes are driven by pollution rather than weather effects, we control for local meteorological variables in our analysis (Chen et al., 2022).

Even though soccer games do not generate as much pollution as industrial activities, endogeneity concerns such as reverse causality, measurement error, and omitted variables still exist in our context. Thermal inversion offers an exogenous variation in pollution levels, helping us isolate the causal impact of air pollution on player performance.

**Figure A1 The Impact of Air Pollution across Different Positions: MBI Framework**

The figures plot the impact of air pollution (AQI, PM2.5, and PM10) for different position subsamples (Defender, Midfielder, and Forward) with 95% confidence intervals using magnitude-based inferences (MBI). The blue diamonds are computed as two times the standardized regression coefficients on air pollution. The vertical lines ($\pm0.2$) indicate the threshold value for non-trivial effect according to MBI (<0.2 trivial; 0.2-0.6 small; 0.6-1.2 moderate; 1.2-2.0 large; >2.0 very large). The results for number of passes, fouls, and running distances are reported sequentially.


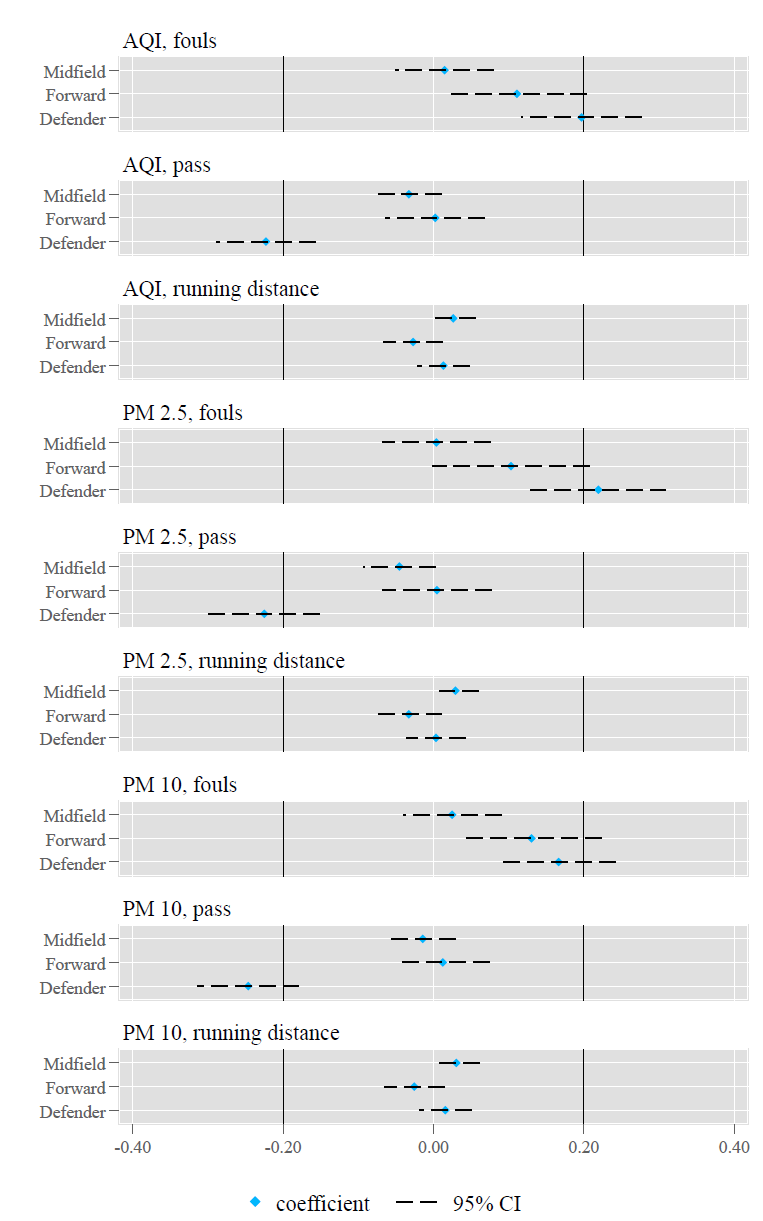


**Table A1 List of CFASL Stadiums**

| This table shows the list of the 24 CFASL stadiums where the 632 matches in our sample took place. | |
| --- | --- |
| No. | Stadium Name |
| 1 | Harbin International Conference and Exhibition Sports Center Stadium |
| 2 | Changchun Economic Development Zone Stadium |
| 3 | Yanji People’s Stadium |
| 4 | Shenyang Olympic Sports Center Stadium |
| 5 | Panjin Jinxiu Stadium |
| 6 | Panjin Stadium |
| 7 | Dalian Sports Center Stadium |
| 8 | Qinhuangdao Olympic Sports Center Stadium |
| 9 | Beijing Workers’ Stadium |
| 10 | Tianjin Olympic Center Stadium |
| 11 | Shijiazhuang Yu Long Stadium |
| 12 | Jinan Olympic Sports Center Stadium |
| 13 | Zhengzhou Sailing Stadium |
| 14 | Zhenjiang Sports Center Stadium |
| 15 | Nanjing Olympic Sports Center Stadium |
| 16 | Shanghai Hongkou Soccer Stadium |
| 17 | Shanghai Jinshan Sports Center Stadium |
| 18 | Shanghai Stadium |
| 19 | Shanghai Yuanshen Sports Center Stadium |
| 20 | Hangzhou Huanglong Sports Center Stadium |
| 21 | Chongqing Olympic Sports Center Stadium |
| 22 | Guiyang Olympic Sports Center Stadium |
| 23 | Guangzhou Tianhe Stadium |
| 24 | Guangzhou Yuexiushan Stadium |

**Table A2 Variable Definition**

| This table presents definitions of the main variables examined in this study. | |
| --- | --- |
| Variable | Definition |
| Pass | The number of player’s passes |
| Foul | The number of player’s fouls |
| Running Distance | Natural logarithm of player’s running distance (m) |
| Goals Scored | Total goals scored |
| Goals Against | Total goals lost |
| Goals Difference | Net goals scored |
| Match Outcome 1 | Lost = 0; Win or Tie = 1 |
| Match Outcome 2 | Lost = 0; Tie = 1; Win = 2 |
| AQI | Air Quality Index of the nearest observation station to the stadium (scaled by 100) |
| PM2.5 | Fine particulate matter of the nearest observation station to the stadium (scaled by 100) |
| PM10 | Inhalable particles of the nearest observation station to the stadium (scaled by 100) |
| Playtime | Individual player’s play time |
| Age | Individual player’s age |
| DF Indicator | One for defender position; Zero otherwise |
| M Indicator | One for midfielder position; Zero otherwise |
| FW Indicator | One for forward position; Zero otherwise |
| Temperature | Real-time temperature of the nearest observation station to the stadium |
| Dewpoint | Real-time dewpoint of the nearest observation station to the stadium |
| Humidity | Real-time humidity of the nearest observation station to the stadium |
| Wind speed | Real-time wind speed of the nearest observation station to the stadium |
| Cloudy Day | One for cloudy day; Zero otherwise |
| Thermal Inversion | One if temperature inversion during the matchday; Zero otherwise |

**Table A3 Summary Statistics**

| This table reports the summary statistics for the main variables examined in this study. | | | | | | | | |
| --- | --- | --- | --- | --- | --- | --- | --- | --- |
| Variable | Obs. | Mean | Std. | Min | p25 | p50 | p75 | Max |
| Pass | 15,834 | 28.009 | 17.498 | 0 | 15 | 26 | 38 | 132 |
| Foul | 15,834 | 1.294 | 1.336 | 0 | 0 | 1 | 2 | 11 |
| Running Distance | 15,831 | 8.836 | 0.736 | 2.833 | 8.756 | 9.149 | 9.249 | 9.492 |
| Goals Scored | 1,244 | 1.392 | 1.21 | 0 | 0 | 1 | 2 | 7 |
| Goals Against | 1,244 | 1.392 | 1.21 | 0 | 0 | 1 | 2 | 7 |
| Goals Difference | 1,244 | 1.392 | 1.21 | 0 | 0 | 1 | 2 | 7 |
| Match Outcome 1 | 1,244 | 0.639 | 0.48 | 0 | 0 | 1 | 1 | 1 |
| Match Outcome 2 | 1,244 | 1 | 0.85 | 0 | 0 | 1 | 2 | 2 |
| AQI | 15,733 | 0.749 | 0.509 | 0.15 | 0.42 | 0.62 | 0.9 | 5 |
| PM2.5 | 15,681 | 0.467 | 0.398 | 0.01 | 0.22 | 0.36 | 0.6 | 3.67 |
| PM10 | 14,895 | 0.838 | 0.696 | 0 | 0.4 | 0.63 | 1.04 | 7.73 |
| Playtime | 15,832 | 74.959 | 30.092 | 0 | 56 | 94 | 96 | 104 |
| Age | 15,834 | 28.421 | 3.664 | 17 | 26 | 28 | 31 | 39 |
| DF Indicator | 15,834 | 0.341 | 0.474 | 0 | 0 | 0 | 1 | 1 |
| M Indicator | 15,834 | 0.455 | 0.498 | 0 | 0 | 0 | 1 | 1 |
| FW Indicator | 15,834 | 0.204 | 0.403 | 0 | 0 | 0 | 0 | 1 |
| Temperature | 15,732 | 21.853 | 6.693 | -3 | 18 | 23 | 27 | 35 |
| Dewpoint | 15,226 | 15.061 | 8.985 | -20 | 11 | 17 | 22 | 27 |
| Humidity | 15,226 | 67.111 | 21.289 | 8 | 56 | 70 | 83 | 100 |
| Wind speed | 15,732 | 10.915 | 6.477 | 0 | 7.2 | 10.8 | 14.4 | 39.6 |
| Cloudy Day | 15,834 | 0.347 | 0.476 | 0 | 0 | 0 | 1 | 1 |
| Thermal Inversion | 15,834 | 0.416 | 0.493 | 0 | 0 | 0 | 1 | 1 |

**Table A4 First-stage estimation: Effects of Thermal Inversion on Air Pollution Indicators**

| This table reports the results of the first-stage IV estimation. The dependent variables are AQI (Columns 1-3), PM2.5 (Column 4), and PM10 (Column 5). The independent variable is thermal inversion, which indicates whether a temperature inversion happened in the matchday. We also control for player characteristics and weather variables. Standard errors are clustered at the player-level and reported in parentheses. * p < 0.1, ** p < 0.05, *** p < 0.01. | | | | | |
| --- | --- | --- | --- | --- | --- |
|  | AQI | AQI | AQI | PM2.5 | PM10 |
|  | (1) | (2) | (3) | (4) | (5) |
| Thermal Inversion | 0.309^***^ | 0.312^***^ | 0.314^***^ | 0.243^***^ | 0.387^***^ |
|  | (0.008) | (0.008) | (0.008) | (0.007) | (0.010) |
| Player and Weather Controls | Y | Y | Y | Y | Y |
| Player FE | Y | Y | Y | Y | Y |
| Home FE | Y | Y | Y | Y | Y |
| Team FE | N | Y | N | N | N |
| Season FE | N | Y | N | N | N |
| Team*Season FE | N | N | Y | Y | Y |
| KP F-statistic | 528.461 | 524.492 | 518.023 | 409.202 | 570.350 |
| Observations | 15061 | 15061 | 15061 | 15009 | 14227 |

“FE” stands for fixed effect.

**Table A5 Impact of Air Pollution on Soccer Players’ Performance: Passes and Fouls (Standardized Coefficients)**

| This table shows the standardized regression coefficients for the results in Table 1, which are obtained by first standardizing all the relevant variables before running the regression. The standard errors are clustered at the player-level and reported in parentheses. * p < 0.1, ** p < 0.05, *** p < 0.01. | | | | | | | | | | | |
| --- | --- | --- | --- | --- | --- | --- | --- | --- | --- | --- | --- |
|  | Panel A: No. of Passes | | | | | Panel B: No. of Fouls | | | | | |
|  | (1) | (2) | (3) | (4) | (5) | | (6) | (7) | (8) | (9) | (10) |
| AQI | -0.035** | -0.038** | -0.040** |  |  | | 0.041^*^ | 0.041^*^ | 0.046^**^ |  |  |
|  | (0.017) | (0.016) | (0.016) |  |  | | (0.023) | (0.022) | (0.022) |  |  |
| PM2.5 |  |  |  | -0.044** |  | |  |  |  | 0.046^*^ |  |
|  |  |  |  | (0.018) |  | |  |  |  | (0.025) |  |
| PM10 |  |  |  |  | -0.039** | |  |  |  |  | 0.049^**^ |
|  |  |  |  |  | (0.016) | |  |  |  |  | (0.022) |
| Player and Weather Controls | Y | Y | Y | Y | Y | | Y | Y | Y | Y | Y |
| Player FE | Y | Y | Y | Y | Y | | Y | Y | Y | Y | Y |
| Home FE | Y | Y | Y | Y | Y | | Y | Y | Y | Y | Y |
| Team FE | N | Y | N | N | N | | N | Y | N | N | N |
| Season FE | N | Y | N | N | N | | N | Y | N | N | N |
| Team*Season FE | N | N | Y | Y | Y | | N | N | Y | Y | Y |
| Observations | 15061 | 15061 | 15061 | 15009 | 14227 | | 15061 | 15061 | 15061 | 15009 | 14227 |

“FE” stands for fixed effect.

**Table A6 Impact of Air Pollution on Soccer Players’ Performance: Passes and Fouls (Player Random Effect)**

| This table reports the results of air pollution’s impact on player’s passes and fouls based on a mixed model that assumes random player identities. Columns (1)-(3) show the results for the number of passes while Columns (4)-(6) show the results for the number of fouls. The independent variables are AQI (Columns 1 and 4), PM2.5 (Columns 2 and 5), and PM10 (Columns 3 and 6). We control for player characteristics and weather variables. Standard errors are clustered at the player-level and reported in parentheses. * p < 0.1, ** p < 0.05, *** p < 0.01. | | | | | | | |
| --- | --- | --- | --- | --- | --- | --- | --- |
|  | No. of Passes | | | No. of Fouls | | | |
|  | (1) | (2) | (3) | | (4) | (5) | (6) |
| **Panel A: Original Coefficients** | | | | | | | |
| AQI | -1.379^***^ |  |  | | 0.117^**^ |  |  |
|  | (0.527) |  |  | | (0.058) |  |  |
| PM2.5 |  | -1.925^**^ |  | |  | 0.148^*^ |  |
|  |  | (0.752) |  | |  | (0.0825) |  |
| PM10 |  |  | -0.973^**^ | |  |  | 0.089^**^ |
|  |  |  | (0.3844) | |  |  | (0.0422) |
| **Panel B: Standardized Coefficients** | | | | | | | |
| AQI | -0.041^***^ |  |  | | 0.045^**^ |  |  |
|  | (0.016) |  |  | | (0.022) |  |  |
| PM2.5 |  | -0.044^**^ |  | |  | 0.044^*^ |  |
|  |  | (0.017) |  | |  | (0.025) |  |
| PM10 |  |  | -0.039^**^ | |  |  | 0.047^**^ |
|  |  |  | (0.015) | |  |  | (0.022) |
| Player and Weather Controls | Y | Y | Y | | Y | Y | Y |
| Home FE | Y | Y | Y | | Y | Y | Y |
| Team*Season FE | Y | Y | Y | | Y | Y | Y |
| Observations | 15173 | 15121 | 14335 | | 15173 | 15121 | 14335 |

“FE” stands for fixed effect.

**Table A7 The Heterogeneous Impact of Air Pollution across Different Positions: Passes and Fouls**

| This table reports the results that compare air pollution’s impact across different positions from the second stage IV estimation: defender (Columns 1, 4, and 7), midfield (Columns 2, 5, and 8), and forward (Columns 3, 6, and 9). Panel A reports the results for the number of passes and Panel B the number of fouls. We control for player characteristics and weather variables. Standard errors are clustered at the player-level and reported in parentheses. * p < 0.1, ** p < 0.05, *** p < 0.01. | | | | | | | | | |
| --- | --- | --- | --- | --- | --- | --- | --- | --- | --- |
|  | Defender | Midfield | Forward | Defender | Midfield | Forward | Defender | Midfield | Forward |
|  | (1) | (2) | (3) | (4) | (5) | (6) | (7) | (8) | (9) |
| **Panel A: No. of Passes** |  |  |  |  |  |  |  |  |  |
| AQI | -3.379*** | -0.633 | 0.027 |  |  |  |  |  |  |
|  | (1.017) | (0.863) | (0.760) |  |  |  |  |  |  |
| PM2.5 |  |  |  | -4.450*** | -1.104 | 0.070 |  |  |  |
|  |  |  |  | (1.486) | (1.198) | (1.124) |  |  |  |
| PM10 |  |  |  |  |  |  | -2.698*** | -0.206 | 0.104 |
|  |  |  |  |  |  |  | (0.753) | (0.642) | (0.535) |
| **Panel B: No. of Fouls** |  |  |  |  |  |  |  |  |  |
| AQI | 0.244** | 0.019 | 0.160 |  |  |  |  |  |  |
|  | (0.101) | (0.084) | (0.137) |  |  |  |  |  |  |
| PM2.5 |  |  |  | 0.355** | 0.006 | 0.194 |  |  |  |
|  |  |  |  | (0.148) | (0.117) | (0.199) |  |  |  |
| PM10 |  |  |  |  |  |  | 0.158** | 0.023 | 0.135 |
|  |  |  |  |  |  |  | (0.073) | (0.063) | (0.099) |
| Player and Weather Controls | Y | Y | Y | Y | Y | Y | Y | Y | Y |
| Player FE | Y | Y | Y | Y | Y | Y | Y | Y | Y |
| Home FE | Y | Y | Y | Y | Y | Y | Y | Y | Y |
| Team*Season FE | Y | Y | Y | Y | Y | Y | Y | Y | Y |
| Observations | 5067 | 6937 | 3056 | 5048 | 6912 | 3048 | 4798 | 6530 | 2898 |

“FE” stands for fixed effect.

**Table A8 The Heterogeneous Impact of Air Pollution across Different Positions: Passes and Fouls (Standardized Coefficients)**

| This table shows the standardized regression coefficients for the results in Table A7, which are obtained by first standardizing all the relevant variables before running the regression. The standard errors are clustered at the player-level and reported in parentheses. * p < 0.1, ** p < 0.05, *** p < 0.01. | | | | | | | | | |
| --- | --- | --- | --- | --- | --- | --- | --- | --- | --- |
|  | Defender | Midfield | Forward | Defender | Midfield | Forward | Defender | Midfield | Forward |
|  | (1) | (2) | (3) | (4) | (5) | (6) | (7) | (8) | (9) |
| **Panel A: No. of Passes** |  |  |  |  |  |  |  |  |  |
| AQI | -0.111^***^ | -0.016 | 0.001 |  |  |  |  |  |  |
|  | (0.034) | (0.022) | (0.034) |  |  |  |  |  |  |
| PM2.5 |  |  |  | -0.113^***^ | -0.023 | 0.002 |  |  |  |
|  |  |  |  | (0.038) | (0.025) | (0.037) |  |  |  |
| PM10 |  |  |  |  |  |  | -0.123^***^ | -0.007 | 0.006 |
|  |  |  |  |  |  |  | (0.034) | (0.022) | (0.032) |
| **Panel B: No. of Fouls** |  |  |  |  |  |  |  |  |  |
| AQI | 0.099^**^ | 0.007 | 0.056 |  |  |  |  |  |  |
|  | (0.041) | (0.033) | (0.047) |  |  |  |  |  |  |
| PM2.5 |  |  |  | 0.110^**^ | 0.002 | 0.052 |  |  |  |
|  |  |  |  | (0.046) | (0.036) | (0.053) |  |  |  |
| PM10 |  |  |  |  |  |  | 0.088^**^ | 0.012 | 0.065 |
|  |  |  |  |  |  |  | (0.041) | (0.033) | (0.048) |
| Player and Weather Controls | Y | Y | Y | Y | Y | Y | Y | Y | Y |
| Player FE | Y | Y | Y | Y | Y | Y | Y | Y | Y |
| Home FE | Y | Y | Y | Y | Y | Y | Y | Y | Y |
| Team*Season FE | Y | Y | Y | Y | Y | Y | Y | Y | Y |
| Observations | 5067 | 6937 | 3056 | 5048 | 6912 | 3048 | 4798 | 6530 | 2898 |

“FE” stands for fixed effect.

**Table A9 The Heterogeneous Impact of Air Pollution b/w Home and Away Matches: Passes and Fouls**

| This table reports the results that compare air pollution’s impact between home (Columns 1, 3, and 5) and away (Columns 2, 4, and 6) matches from the second stage IV estimation. Panel A reports the results for the number of passes and Panel B the number of fouls. We control for player characteristics and weather variables. Standard errors are clustered at the player-level and reported in parentheses. * p < 0.1, ** p < 0.05, *** p < 0.01. | | | | | | |
| --- | --- | --- | --- | --- | --- | --- |
|  | Home | Away | Home | Away | Home | Away |
|  | (1) | (2) | (3) | (4) | (5) | (6) |
| **Panel A: No. of Passes** | | | | | | |
| AQI | -0.705 | -1.728^**^ |  |  |  |  |
|  | (1.144) | (0.7424 |  |  |  |  |
| PM2.5 |  |  | -0.970 | -2.586^**^ |  |  |
|  |  |  | (1.482) | (1.045) |  |  |
| PM10 |  |  |  |  | -0.391 | -1.117^**^ |
|  |  |  |  |  | (0.963) | (0.550) |
| **Panel B: No. of Fouls** | | | | | | |
| AQI | 0.040 | 0.180^*^ |  |  |  |  |
|  | (0.114) | (0.096) |  |  |  |  |
| PM2.5 |  |  | 0.058 | 0.242^*^ |  |  |
|  |  |  | (0.148) | (0.138) |  |  |
| PM10 |  |  |  |  | 0.021 | 0.144^**^ |
|  |  |  |  |  | (0.099 | (0.070) |
| Player and Weather Controls | Y | Y | Y | Y | Y | Y |
| Player FE | Y | Y | Y | Y | Y | Y |
| Home FE | Y | Y | Y | Y | Y | Y |
| Team*Season FE | Y | Y | Y | Y | Y | Y |
| Observations | 7479 | 7488 | 7453 | 7462 | 7060 | 7071 |

“FE” stands for fixed effect.

**Table A10 The Heterogeneous Impact of Air Pollution b/w Home and Away Matches: Passes and Fouls (Standardized Coefficients)**

| This table shows the standardized regression coefficients for the results in Table A9, which are obtained by first standardizing all the relevant variables before running the regression. The standard errors are clustered at the player-level and reported in parentheses. * p < 0.1, ** p < 0.05, *** p < 0.01. | | | | | | |
| --- | --- | --- | --- | --- | --- | --- |
|  | Home | Away | Home | Away | Home | Away |
|  | (1) | (2) | (3) | (4) | (5) | (6) |
| **Panel A: No. of Passes** | | | | | | |
| AQI | -0.020 | -0.053^**^ |  |  |  |  |
|  | (0.033) | (0.023) |  |  |  |  |
| PM2.5 |  |  | -0.022 | -0.062^**^ |  |  |
|  |  |  | (0.033) | (0.025) |  |  |
| PM10 |  |  |  |  | -0.015 | -0.0470^**^ |
|  |  |  |  |  | (0.037) | (0.023) |
| *N* | 7479 | 7488 | 7453 | 7462 | 7060 | 7071 |
| **Panel B: No. of Fouls** | | | | | | |
| AQI | 0.016 | 0.068^*^ |  |  |  |  |
|  | (0.044) | (0.036) |  |  |  |  |
| PM2.5 |  |  | 0.018 | 0.071^*^ |  |  |
|  |  |  | (0.045) | (0.040) |  |  |
| PM10 |  |  |  |  | 0.011 | 0.075^**^ |
|  |  |  |  |  | (0.052) | (0.036) |
| Player and Weather Controls | Y | Y | Y | Y | Y | Y |
| Player FE | Y | Y | Y | Y | Y | Y |
| Home FE | Y | Y | Y | Y | Y | Y |
| Team*season FE | Y | Y | Y | Y | Y | Y |
| Observations | 7479 | 7488 | 7453 | 7462 | 7060 | 7071 |

“FE” stands for fixed effect.

**Table A11 Non-linearity in Air Pollution’s Impact**

| This table reports the results examining the non-linear effect of air pollution. We first exploit the Kernel-weighted local polynomial smoothing (without further control variables) to determine the different threshold levels of air pollution measures (AQI, PM2.5, and PM10). Based on these threshold levels, we generate two sets of pollution dummies: Intermediate Pollution Level and High Pollution Level, which are used as the key independent variables in the OLS regression analysis. Columns (1)-(3) show the results for the number of passes while Columns (4)-(16) show the results for the number of fouls. We control for player characteristics and weather variables. Standard errors are clustered at the player-level and reported in parentheses. * p < 0.1, ** p < 0.05, *** p < 0.01. | | | | | | |
| --- | --- | --- | --- | --- | --- | --- |
|  | No. of Passes | | | No. of Fouls | | |
|  | AQI | PM2.5 | PM10 | AQI | PM2.5 | PM10 |
|  | (1) | (2) | (3) | (4) | (5) | (6) |
| Intermediate Pollution Level | -0.562*** | -0.302* | -0.573*** | 0.050* | 0.054* | 0.088* |
|  | (0.197) | (0.178) | (0.215) | (0.028) | (0.031) | (0.047) |
| High Pollution Level | -0.742** | -0.712** | -1.180*** | 0.064* | 0.107** | 0.106** |
|  | (0.359) | (0.342) | (0.282) | (0.037) | (0.042) | (0.053) |
| Player and Weather Controls | Y | Y | Y | Y | Y | Y |
| Player FE | Y | Y | Y | Y | Y | Y |
| Home FE | Y | Y | Y | Y | Y | Y |
| Team*Season FE | Y | Y | Y | Y | Y | Y |
| Observations | 15112 | 15112 | 15112 | 15112 | 15112 | 15112 |

“FE” stands for fixed effect.

**Table A12 Impact of Air Pollution on Soccer Players’ Performance: Running Distances (Additional Analysis)**

| Panel A of this table reports the results that compare air pollution’s impact on running distances across different positions: defender (Columns 1, 4, and 7), midfield (Columns 2, 5, and 8), and forward (Columns 3, 6, and 9). Panel B reports the results that compare air pollution’s impact between home (Columns 1, 3, and 5) and away (Columns 2, 4, and 6) matches. Panel C reports the results examining the non-linear effect of air pollution: we first exploit the Kernel-weighted local polynomial smoothing (without further control variables) to determine the different threshold levels of air pollution measures (AQI, PM2.5, and PM10). Based on these threshold levels, we generate two sets of pollution dummies: Intermediate Pollution Level and High Pollution Level, which are used as the key independent variables in the OLS regression analysis. We control for player characteristics and weather variables. Standard errors are clustered at the player-level and reported in parentheses. * p < 0.1, ** p < 0.05, *** p < 0.01. | |
| --- | --- |
|  | Running Distances (Player and Weather Controls: Y; Player FE: Y; Home FE: Y; Team*Season FE: Y) |

| **Panel A: Positions** | Defender | Midfield | Forward | Defender | Midfield | Forward | Defender | Midfield | Forward |
| --- | --- | --- | --- | --- | --- | --- | --- | --- | --- |
|  | (1) | (2) | (3) | (4) | (5) | (6) | (7) | (8) | (9) |
| AQI | 0.008 | 0.020 | -0.019 |  |  |  |  |  |  |
|  | (0.021) | (0.023) | (0.029) |  |  |  |  |  |  |
| PM2.5 |  |  |  | 0.003 | 0.029 | -0.030 |  |  |  |
|  |  |  |  | (0.030) | (0.031) | (0.042) |  |  |  |
| PM10 |  |  |  |  |  |  | 0.007 | 0.018 | -0.013 |
|  |  |  |  |  |  |  | (0.015) | (0.018) | (0.021) |
| Observations | 5065 | 6936 | 3056 | 5046 | 6911 | 3048 | 4796 | 6529 | 2898 |

| **Panel B: Home vs. Away** | Home | Away | Home | Away | Home | Away |
| --- | --- | --- | --- | --- | --- | --- |
|  | (1) | (2) | (3) | (4) | (5) | (6) |
| AQI | 0.038 | -0.016 |  |  |  |  |
|  | (0.027) | (0.020) |  |  |  |  |
| PM2.5 |  |  | 0.049 | -0.024 |  |  |
|  |  |  | (0.035) | (0.029) |  |  |
| PM10 |  |  |  |  | 0.033 | -0.011 |
|  |  |  |  |  | (0.023) | (0.015) |
| Observations | 7477 | 7487 | 7451 | 7461 | 7058 | 7070 |

| **Panel C: Non-Linearity** | AQI | PM2.5 | PM10 |
| --- | --- | --- | --- |
|  | (1) | (2) | (3) |
| Intermediate Pollution Level | 0.002 | -0.007 | -0.013** |
|  | (0.005) | (0.007) | (0.006) |
| High Pollution Level | -0.029 | -0.002 | -0.003 |
|  | (0.018) | (0.008) | (0.006) |
| Observations | 15109 | 15109 | 15109 |

**Table A13 Impact of Air Pollution on Match Outcome: No. of Goals**

| This table reports the results of air pollution’s impact on the number of goals from the second stage of match-level IV estimation. We report results separately for goals scored (Columns 1-3), goals against (Columns 4-6), and goal difference (Columns 7-8). The main variable of interest is the interaction term between different air pollution measures (AQI (Columns 1, 4, and 7), PM2.5 (Columns 2, 5, and 8), and PM10 (Columns 3, 6, and 9)) and the away match indicator. We control for team characteristics and weather variables. Standard errors are clustered at the player-level and reported in parentheses. * p < 0.1, ** p < 0.05, *** p < 0.01. | | | | | | | | | |
| --- | --- | --- | --- | --- | --- | --- | --- | --- | --- |
|  | Goals Scored | | | Goals Against | | | Goals Difference | | |
|  | (1) | (2) | (3) | (4) | (5) | (6) | (7) | (8) | (9) |
| AQI $\times$ Away | -0.843^**^ |  |  | 0.694^*^ |  |  | -1.537^***^ |  |  |
|  | (0.402) |  |  | (0.349) |  |  | (0.503) |  |  |
| AQI | 0.239 |  |  | -0.560 |  |  | 0.799 |  |  |
|  | (0.379) |  |  | (0.341) |  |  | (0.541) |  |  |
| PM25 $\times$ Away |  | -1.376^**^ |  |  | 1.022^*^ |  |  | -2.398^***^ |  |
|  |  | (0.583) |  |  | (0.561) |  |  | (0.830) |  |
| PM25 |  | 0.337 |  |  | -0.897^*^ |  |  | 1.234 |  |
|  |  | (0.518) |  |  | (0.509) |  |  | (0.791) |  |
| PM10 $\times$ Away |  |  | -0.564^*^ |  |  | 0.498^*^ |  |  | -1.062^***^ |
|  |  |  | (0.305) |  |  | (0.241) |  |  | (0.370) |
| PM10 |  |  | 0.238 |  |  | -0.353 |  |  | 0.591 |
|  |  |  | (0.277) |  |  | (0.255) |  |  | (0.384) |
| Away | 0.259 | 0.274 | 0.147 | -0.139 | -0.097 | -0.073 | 0.398 | 0.371 | 0.219 |
|  | (0.312) | (0.280) | (0.231) | (0.263) | (0.262) | (0.194) | (0.391) | (0.398) | (0.291) |
| Team and Weather Controls | Y | Y | Y | Y | Y | Y | Y | Y | Y |
| Team*Season FE | Y | Y | Y | Y | Y | Y | Y | Y | Y |
| Observations | 1192 | 1188 | 1126 | 1192 | 1188 | 1126 | 1192 | 1188 | 1126 |

**Table A14 Impact of Air Pollution on Match Outcome: Winning Probability (Binary Outcome)**

| This table reports the results of air pollution’s impact on winning probabilities from the second stage match-level IV estimation. The dependent variable (Match Outcome 1) is an indicator which equals to zero if the team lost the game, and one otherwise. We report results both for the linear probability model (Columns 1-3) and the probit model (Columns 4-6). The main variable of interest is the interaction term between different air pollution measures (AQI (Columns 1 and 4), PM2.5 (Columns 2 and 5), and PM10 (Columns 3 and 6) and the away match indicator. We control for team characteristics and weather variables. Standard errors are clustered at the player-level and reported in parentheses. * p < 0.1, ** p < 0.05, *** p < 0.01. | | | | | | |
| --- | --- | --- | --- | --- | --- | --- |
|  | Match Outcome 1 | | | | | |
|  | Linear Probability Model | | | Probit Model | | |
|  | (1) | (2) | (3) | (4) | (5) | (6) |
| AQI $\times$ Away | -0.407** |  |  | -1.15** |  |  |
|  | (0.189) |  |  | (0.518) |  |  |
| AQI | 0.182 |  |  | 0.531 |  |  |
|  | (0.144) |  |  | (0.441) |  |  |
| PM2.5 $\times$ Away |  | -0.582* |  |  | -1.664** |  |
|  |  | (0.298) |  |  | (0.793) |  |
| PM2.5 |  | 0.255 |  |  | 0.76 |  |
|  |  | (0.207) |  |  | (0.621) |  |
| PM10 $\times$ Away |  |  | -0.289* |  |  | -0.805** |
|  |  |  | (0.146) |  |  | (0.394) |
| PM10 |  |  | 0.126 |  |  | 0.357 |
|  |  |  | (0.109) |  |  | (0.324) |
| Away | 0.143 | 0.11 | 0.096 | 0.399 | 0.318 | 0.753 |
|  | (0.139) | (0.138) | (0.119) | (0.406) | (0.395) | (1.237) |
| Team and Weather Controls | Y | Y | Y | Y | Y | Y |
| Team*Season FE | Y | Y | Y | Y | Y | Y |
| Observations | 1192 | 1188 | 1126 | 1192 | 1188 | 1126 |

**Table A15 Impact of Air Pollution on Match Outcome: Winning Probability (Ordered Probit Model)**

| This table reports the results of air pollution’s impact on winning probabilities from the second stage match-level IV estimation. We implement an ordered probit model where the match outcome (Match Outcome 2) contains three values: zero (lose), one (tie), and two (win). The main variable of interest is the interaction term between different air pollution measures (AQI (Column 1), PM2.5 (Column 2), and PM10 (Column 3)) and the away match indicator. We control for team characteristics and weather variables. Standard errors are clustered at the player-level and reported in parentheses. * p < 0.1, ** p < 0.05, *** p < 0.01. | | | |
| --- | --- | --- | --- |
|  | Match Outcome 2 | | |
|  | (1) | (2) | (3) |
| AQI $\times$ Away | -1.124*** |  |  |
|  | (0.41) |  |  |
| AQI | 0.607 |  |  |
|  | (0.373) |  |  |
| PM25 $\times$ Away |  | -1.64** |  |
|  |  | (0.655) |  |
| PM25 |  | 0.841 |  |
|  |  | (0.53) |  |
| PM10 $\times$ Away |  |  | -0.806*** |
|  |  |  | (0.292) |
| PM10 |  |  | 0.472* |
|  |  |  | (0.265) |
| Away | 0.386 | 0.312 | 0.268 |
|  | (0.343) | (0.346) | (0.265) |
| Team and Weather Controls | Y | Y | Y |
| Team*Season FE | Y | Y | Y |
| Observations | 1192 | 1188 | 1126 |

**Reference**

Ali A. Measuring soccer skill performance: a review. Scandinavian Journal of Medicine & Science in Sports, 2011, 21(2): 170-183.

Arceo E, Rema H, Paulina O. Does the effect of pollution on infant mortality differ between developing and developed countries? Evidence from Mexico City. The Economic Journal, 2016, 126 (591): 257–280.

Carling C. Interpreting physical performance in professional soccer match-play: should we be more pragmatic in our approach? Sports Medicine, 2013, 43: 655-663.

Carling C, Dupont G. Are declines in physical performance associated with a reduction in skill-related performance during professional soccer match-play? Journal of Sports Sciences, 2011, 29(1): 63-71.

Chen S, Oliva P, Zhang P. The effect of air pollution on migration: Evidence from China. Journal of Development Economics, 2022, 156: 102833.

Deryugina T, Heutel G, Miller N H, et al. The mortality and medical costs of air pollution: Evidence from changes in wind direction. American Economic Review, 2019, 109(12): 4178-4219.

Dolci F, Hart N H, Kilding A E, et al. Physical and energetic demand of soccer: a brief review. Strength & Conditioning Journal, 2020, 42(3): 70-77.

Fu S, Viard V B, Zhang P. Air pollution and manufacturing firm productivity: Nationwide estimates for China. The Economic Journal, 2021, 131(640): 3241-3273.

Gabbett T J, Carius J, Mulvey M. Does improved decision-making ability reduce the physiological demands of game-based activities in field sport athletes? The Journal of Strength & Conditioning Research, 2008, 22(6): 2027-2035.

Godzinski A, Castillo M S. Disentangling the effects of air pollutants with many instruments. Journal of Environmental Economics and Management, 2021, 109: 102489.

Junge A, Dvorak J, Rosch D, et al. Psychological and sport-specific characteristics of football players. The American Journal of Sports Medicine, 2000, 28(5_suppl): 22-28.

Locke S L. Estimating the impact of major league baseball games on local air pollution. Contemporary Economic Policy, 2019, 37(2): 236–244.

Romeas T, Guldner A, Faubert J. 3D-Multiple Object Tracking training task improves passing decision-making accuracy in soccer players. Psychology of Sport and Exercise, 2016, 22: 1-9.

Slimani M, Bragazzi N L, Tod D, et al. Do cognitive training strategies improve motor and positive psychological skills development in soccer players? Insights from a systematic review. Journal of Sports Sciences, 2016, 34(24): 2338-2349.

Volkamer M. Zurur aggressivitat in Konkumenz—orientierten sozialen. Sport Wissenschaft, 1971, 1: 68-76.

van Maarseveen M J J, Oudejans R R D, Mann D L, et al. Perceptual-cognitive skill and the in situ performance of soccer players. Quarterly Journal of Experimental Psychology, 2018, 71(2): 455-470.

Wan L, Liu Y, Lv K, et al. Firm Innovation in Hazy Days: Chinese Evidence. Environmental and Resource Economics, 2024: 1-32.

Wicker P. The carbon footprint of active sport tourists: An empirical analysis of skiers and boarders. Journal of Sport & Tourism, 2018, 22(2): 151–171.

Wicker P. The carbon footprint of active sport participants. Sport Management Review, 2019, 22(4): 513–526.
